# Prolonged Duration of Blood Pressure Drops During General Anesthesia Is Associated With Worse Outcomes After Mechanical Thrombectomy

**DOI:** 10.3389/fneur.2021.640841

**Published:** 2021-03-29

**Authors:** Chao Xu, Gaoping Lin, Zheyu Zhang, Tianyu Jin, Ning Li, Hui Mao, Sasa Ye, Zongming Yang, Yu Geng, Zongjie Shi

**Affiliations:** ^1^Department of Neurology, Zhejiang Provincial People's Hospital, Hangzhou, China; ^2^Department of Neurology, Zhejiang Chinese Medical University, Hangzhou, China; ^3^Department of Anesthesiology, Zhejiang Provincial People's Hospital, Hangzhou, China; ^4^Department of Radiology, Zhejiang Provincial People's Hospital, Hangzhou, China; ^5^Department of Epidemiology and Biostatistics, Zhejiang University School of Public Health, Hangzhou, China

**Keywords:** blood pressure, mechanical thrombectomy, large artery occlusion, general anesthesia, outcome

## Abstract

**Background and Purpose:** Optimal periprocedural management of blood pressure during mechanical thrombectomy (MT) remains controversial. This study aimed to investigate the relationship between the duration of blood pressure drops during general anesthesia and the outcomes in large vessel occlusion (LVO) patients treated with MT.

**Methods:** We retrospectively reviewed our prospectively collected data for LVO patients treated with MT between January 2018 and July 2020. Intraprocedural mean arterial pressure (MAP) was recorded every 5 min throughout the procedure. Baseline MAP minus each MAP value recorded during general anesthesia was defined ΔMAP. Cumulated time (in min) and longest continuous episode (in min) with ΔMAP more than 10, 15, 20, 25, and 30 mmHg were calculated, respectively. Poor outcome was defined as 90-day modified Rankin score (mRS) 3–6. Associations between cumulated time of different ΔMAP thresholds and poor outcome were determined using binary logistic regression models.

**Results:** A total of 131 patients were finally included in the study. After controlling for age, atrial fibrillation, baseline NIHSS, baseline ASPECTS, procedure duration of MT, and times of retrieval attempts, the results indicated that cumulated time of MAP drop more than 10 mmHg (OR 1.013; 95% CI 1.004–1.023; *P* = 0.007) and 15 mmHg (OR 1.011; 95% CI 1.002–1.020; *P* = 0.017) were independently associated with poor outcomes.

**Conclusion:** Prolonged episodes of intraprocedural MAP lowering were more likely to have poor outcomes in LVO patients following MT with general anesthesia, which might be helpful in guiding intraprocedural hemodynamic management of patients under general anesthesia.

## Introduction

Reperfusion therapy with mechanical thrombectomy (MT) has been a standard treatment for patients with acute ischemic stroke (AIS) due to large vessel occlusion (LVO) (1). Nevertheless, over 50% of patients still experience unfavorable outcome (2–4), whereas factors determining the outcomes have not been fully appreciated. Among modifiable factors that affect outcome, blood pressure (BP) control remains a potential target to improve outcome in LVO patients treated with MT. However, data regarding guidance for the optimal peri-procedural management of BP during MT remain uncertain.

Previous studies have found that AIS patients undergoing MT under general anesthesia may be associated with worse outcomes, possibly because of the increased incidence and severity of BP drop during the procedure (5, 6). Theoretically, following LVO, the fate of the ischemic penumbra mainly depends on the ability to maintain perfusion above the threshold for infarction before recanalization (7, 8). Recently, several studies have demonstrated that a drop in BP during MT under general anesthesia is linked with worse outcomes (9–11). In addition, a recent *post-hoc* analysis of randomized controlled trials found that critical BP thresholds and durations during MT, with MAP <70 mmHg for more than 10 min, are linked with poor outcomes (12). Given individualized baseline BP difference, it may be more reasonable to use BP drops than the absolute value of BP.

In view of these considerations, we aimed to investigate the relationship between duration of BP drop during general anesthesia and outcome in LVO patients undergoing MT and hypothesized that patients with prolonged episodes of BP drops were more likely to have worse outcomes.

## Methods and Materials

### Ethics Statement

The human ethics committee of Zhejiang Provincial People's Hospital approved the protocol of this study. All clinical investigations were conducted according to the principles expressed in the Declaration of Helsinki. All subjects had given written informed consent prior to the study.

### Study Subjects

We reviewed our prospectively collected data for consecutive AIS patients with LVO who received MT between January 2018 and July 2020. We enrolled patients who (1) had CT angiography (CTA)-confirmed LVOs presenting within 6 h of symptom onset, including the internal carotid artery, the middle cerebral artery (M1 or M2), and the basilar artery. Patients presenting at the hospital 6–16 h after symptom onset were included if they met the criteria described in the DEFUSE-3 trial (13). Patients presenting 6–24 h after symptom onset were included if they met the related criteria described in the DAWN trial (14). We also enrolled patients who (2) received MT under general anesthesia, (3) achieved Thrombolysis in Myocardial Infarction (TIMI) 2b/3 recanalization after the procedure, and (4) had modified Rankin Scale (mRS) score at 90 days.

### Baseline Characteristics

We retrieved demographic, clinical, laboratory, and radiological data including age, sex, baseline National Institutes of Health Stroke Scale (NIHSS) score, baseline Alberta Stroke Program Early CT Score (ASPECTS), baseline systolic blood pressure (SBP), and diastolic blood pressure (DBP) parameters, as well as comorbid conditions such as history of smoking, hypertension, diabetes mellitus, atrial fibrillation, and congestive heart failure.

### Choice of Anesthesia and Procedure Characteristics

MT under general anesthesia is our center's standard procedure. Only in a few cases when the anesthesiologist could not arrive at the angio-suite in time, MT under conscious sedation was selected. For the purpose of reducing the heterogeneity of study subjects, we excluded patients with conscious sedation. All data from the anesthesiology reports were collected from the beginning of anesthetic induction until awakening. The current guidelines from American Heart Association/American Stroke Association guidelines recommend to maintain BP ≤ 180/105 mmHg during and for 24 h after procedure (1). At our center, we therefore initiate antihypertensive therapy only when intraprocedural BP is higher than 180/105 mmHg recommended by the guidelines. Propofol, remifentanil, and cisatracurium were used to induce general anesthesia. Propofol was used for the maintenance of anesthesia with supplemental dezocine and midazolam as needed. Ephedrine, norepinephrine, and phenylephrine are the medications used to elevate BP during the procedure. Time from onset to reperfusion, general anesthesia duration, and the number of devices passes were also recorded.

### Assessment of Duration of MAP Drop

At our comprehensive stroke center, invasive BP values were recorded every 5 min throughout the procedure. Baseline BP value was defined as the BP measured at admission. The MAP was calculated using the formula MAP = DBP + 1/3(SBP–DBP). Baseline MAP minus each MAP value recorded during general anesthesia was defined as ΔMAP. As our main measure of the different ΔMAP duration during general anesthesia, cumulated time (in min), and longest continuous episode (in min) with ΔMAP more than 10 mmHg, while a cutoff value of 15, 20, 25, and 30 mmHg were also calculated, respectively.

### Evaluation of Outcomes

Degree of recanalization at the end of endovascular procedure was defined by Thrombolysis in Cerebral Infarction (TICI) scores of 2b or 3; a good outcome was defined as mRS score 0–2, and a poor outcome was defined as mRS score 3–6 at 90 days. At 90 days, an outcome assessment was performed with each patient *via* a structured telephone interview by two certified neurologists with clinical experience for 8 and 13 years, respectively (15–18).

### Statistical Analysis

The patients were dichotomized according to the functional outcome. Clinical characteristic and imaging profiles were summarized as mean ± SD or median (25th−75th percentile) for quantitative variables depending on the normality of the distribution and as frequency (percentage) for categorical variables. The Fisher exact test was used to compare the dichotomous variables between two groups, whereas an independent sample 2-tailed *t*-test or a Mann-Whitney *U*-test was used for the continuous variables, depending on the normality of the distribution. Associations of each ΔMAP duration parameter with clinical outcomes were determined using binary logistic regression models adjusted by baseline characteristics with a *P* < 0.1 in univariate analyses, respectively. All statistical analyses were performed using SPSS, Version 22.0 (IBM, Armonk, New York). A *P* < 0.05 was considered statistically significant.

## Results

A total of 131 patients were finally enrolled after excluding patients due to TICI 0–2a after the procedure (*n* = 10), conscious sedation (*n* = 23), and lost to follow-up (*n* = 1). Of the patients included, the mean age was 67.2 ± 13.0 years, and 46 (35.1%) were female. The median NIHSS score on admission was 19 (interquartile range, 15–24), mean time from onset to reperfusion was 460.4 ± 206.1 min and median procedure duration was 124.5 ± 59.2 min.

### Duration of MAP Drops and Functional Outcomes

The MAP at admission was 105.4 ± 12.2 mmHg. Intraprocedural mean MAP was 87.6 ± 12.1 mmHg. Compared with their own admission BP, 115 (87.7%) of patients experienced a drop in MAP during general anesthesia. The mean MAP drop (ΔMAP) was 19.5 ± 14.1 mmHg.

As shown in Table 1, patients with poor outcome were older (70.4 vs. 63.5 years, *P* = 0.002), had a higher frequency of atrial fibrillation (60.6 vs. 36.7%, *P* = 0.006), higher baseline NIHSS score (22 vs. 16, *P* < 0.001), smaller baseline ASPECTS (8 vs. 10, *P* < 0.001), longer procedure duration (134.2 vs. 113.1 min, *P* = 0.038), and underwent more retrieval attempts (2 vs. 1, *P* = 0.002) than those with good outcome.

**Table 1:** Comparison of characteristics between patients with good and poor outcome.

	**Good outcome (*n* = 60)**	**Poor outcome (*n* = 71)**	***P-*value**
Age (year), mean ± SD	63.5 ± 13.3	70.4 ± 12.0	0.002^*^
Female, *n* (%)	18 (30.0)	28 (39.4)	0.260
**Comorbid conditions**			
Smoking, *n* (%)	17 (28.3)	13 (18.3)	0.174
Hypertension, *n* (%)	35 (58.3)	47 (66.2)	0.354
Diabetes mellitus, *n* (%)	12 (20.0)	10 (14.1)	0.367
Atrial fibrillation, *n* (%)	22 (36.7)	43 (60.6)	0.006^*^
Congestive heart failure, *n* (%)	20 (33.3)	23 (37.4)	0.909
**Clinical variables**			
Baseline NIHSS, median (IQR)	16 (12–20)	22 (17–26)	<0.001^*^
Baseline MAP (mmHg), mean ± SD	103.7 ± 13.2	106.8 ± 11.2	0.157
Baseline ASPECTS, median (IQR)	10 (9–10)	8 (7–10)	<0.001^*^
Occlusion site, *n* (%)			0.164
ICA	20 (33.3)	34 (47.9)	
M1	28 (46.7)	26 (36.6)	
M2	7 (11.7)	3 (4.2)	
BA	5 (8.3)	8 (11.3)	
**Intraprocedural management**			
Intraprocedural MAP (mmHg), mean ± SD	88.3 ± 12.5	86.9 ± 11.8	0.502
Onset to reperfusion time (min), median (IQR)	446.1 ± 206.0	477.5 ± 206.8	0.466
Procedure duration (min), median (IQR)	113.1 ± 50.2	134.2 ± 64.7	0.038^*^
Times of retrieval attempts, median (IQR)	1 (1–2)	2 (1–3)	0.002^*^
Vasopressor use, *n* (%)	33 (55.0)	44 (62.0)	0.419
**Duration of different MAP drop level under general anesthesia (min), mean** **±** **SD**			
Drop of 10 mmHg or greater	71.0 ± 57.7	110.2 ± 60.8	<0.001^*^
Drop of 15 mmHg or greater	57.3 ± 56.7	93.6 ± 63.4	0.001^*^
Drop of 20 mmHg or greater	43.3 ± 57.5	74.6 ± 63.0	0.004^*^
Drop of 25 mmHg or greater	34.6 ± 54.4	52.8 ± 57.8	0.067
Drop of 30 mmHg or greater	25.5 ± 48.8	37.9 ± 52.1	0.163

NIHSS, National Institute of Health Stroke Scale; MAP, mean arterial pressure; ASPECTS, Alberta Stroke Program Early CT Score; ICA, internal carotid artery; M1, First segment of middle cerebral artery; M2, Second segment of middle cerebral artery; BA, basilar artery; P^*^ indicates statistical significance.

**Figure 1:**
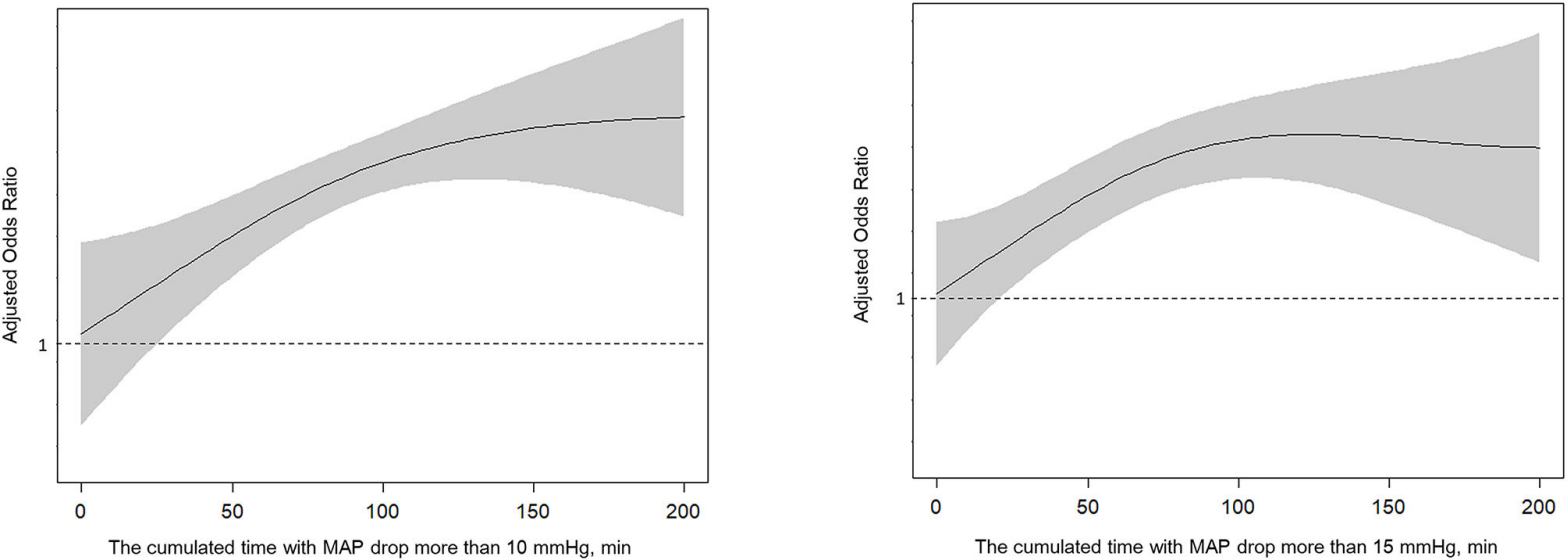
Spline plots of cumulated time (minutes) with MAP drop more than 10 or 15 mmHg and adjusted odds ratio (OR).

**Figure 2:**
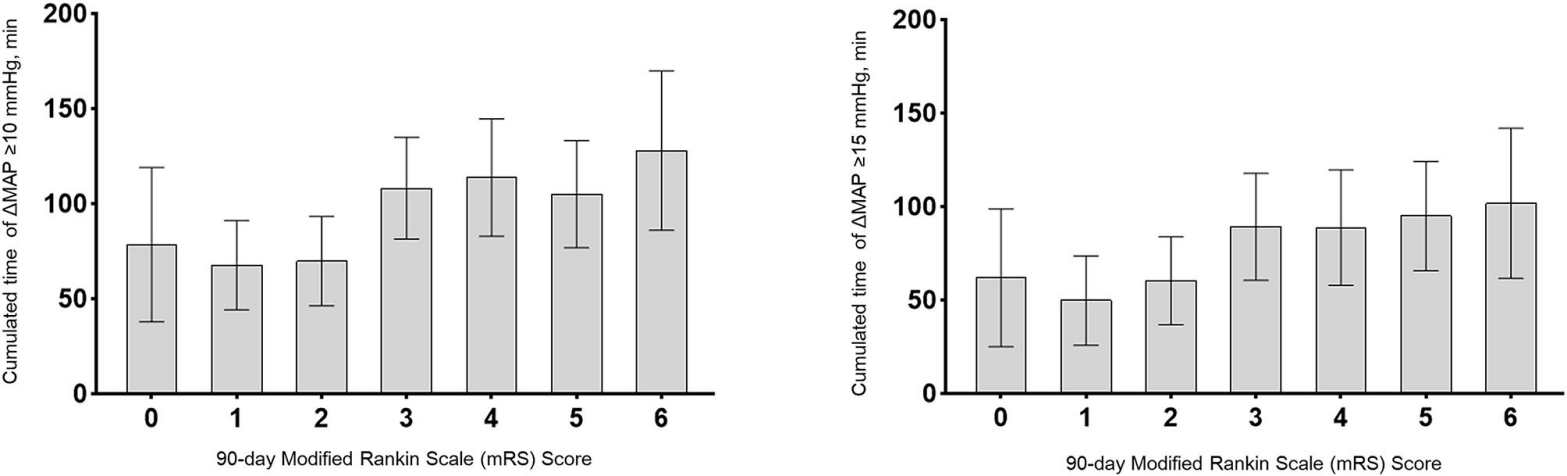
Association of cumulated time (minutes) with functional outcomes at 90 days. Cumulated times with MAP drop more than 10 mmHg or 15 mmHg were plotted per each modified Rankin Scale (mRS) score at 90 days. Bar graphs represent cumulated time with MAP drop for each mRS score category; Error bars indicate the 95% CI.

The cumulated time of MAP dropping more than 10 mmHg (110.2 vs. 71.0 min, *P* < 0.001), the cumulated time of MAP dropping more than 15 mmHg (93.6 vs. 57.3 min, *P* = 0.001), and the cumulated time of MAP dropping more than 20 mmHg (74.6 vs. 43.3 min, *P* = 0.004) were longer in the poor outcome group. There were no significant differences in other variables including baseline BP. After controlling for age, atrial fibrillation, baseline NIHSS, baseline ASPECTS, procedure duration of MT and times of retrieval attempts, we found the results indicated that cumulated time of MAP drop more than 10 mmHg (OR 1.013; 95% CI 1.004–1.023; *P* = 0.007) and 15 mmHg (OR 1.011; 95% CI 1.002–1.020; *P* = 0.017) were independently associated with poor outcomes (Table 2 and Figure 1). For all patients, their score on the mRS at 90 days and their respective cumulated times with MAP drop are shown in Figure 2.

**Table 2:** Binary logistic regression analyses of associations between duration of MAP drop levels under general anesthesia and poor outcome.

	**OR**	**95% CI**	***P*-value**
Drop of 10 mmHg or greater	**1.013**	**1.004–1.023**	**0.007**
Drop of 15 mmHg or greater	**1.011**	**1.002–1.020**	**0.017**
Drop of 20 mmHg or greater	1.007	0.999–1.016	0.076
Drop of 25 mmHg or greater	1.004	0.995–1.012	0.416
Drop of 30 mmHg or greater	1.005	0.995–1.014	0.364

*The associations of each MAP drop level with poor outcome were determined using binary logistic regression models adjusted for age, atrial fibrillation, baseline NIHSS, baseline ASPECTS, procedure duration of MT, and times of retrieval attempts. Bold type indicates statistical significance*.

## Discussion

In this study, our findings suggest that prolonged episodes of lower MAP compared with baseline are related to a higher likelihood of poor outcome in LVO patients following MT with general anesthesia.

A drop in BP during general anesthesia is a common phenomenon in LVO patients receiving MT, which are thought to occur mainly during the induction phase of general anesthesia. The incidence of this phenomenon reported by recent research is about 87% (19). Similarly, we found ~87.7% patients had drop in BP throughout the procedure. Several previous studies indicated that such BP drop during procedures under general anesthesia is inversely correlated with functional outcome. Lowhagen Henden et al. found that a fall in MAP of >40% during endovascular therapy from baseline is an independent predictor of poor neurologic outcomes in LVO patient under general anesthesia (10). Recently, an observational study indicated that patients with an intraprocedural BP drop are likely predisposed to larger infarct volume and worse functional outcome (19). The underlying mechanisms of BP decrease and worse outcomes have not been elucidated to date. The most widely accepted hypothesis is that the ischemic penumbra surrounding the infarct core in patients with LVO has impaired cerebral autoregulation and is sensitive to alterations in systemic blood pressure (20, 21). As a result, hypotension and hemodynamic lability during general anesthesia may overwhelm the brain's ability to autoregulate cerebral blood flow and thereby worsen the extent of injury to the ischemic penumbra and hence worsen functional outcome (19, 22, 23).

Considering that the SBP or DBP variables are more susceptible to be influenced by measurement errors and short BP fluctuations, we thus specifically chose the MAP instead of the SBP or DBP variables. Moreover, in the setting of arterial non-compliance or stiffness, SBP and DBP often move in the opposite direction, especially in an increasingly aging population (24). MAP refers to a combination of systolic and diastolic blood pressures, which is considered a more valid and reliable index and better approximates the perfusion pressure in the brain (25).

Interestingly, we found that only a cutoff value of 10 and 15 mmHg in intraprocedural MAP drop were independently associated with poor outcome. A previous study demonstrated that a fall in MAP of >40%, that is, ~40 mmHg, during endovascular therapy from baseline is an independent predictor of worse functional outcomes in LVO patients under general anesthesia (10). The reason for such a discrepancy may be due to the difference in the definition of baseline MAP. We defined the first recorded MAP value at admission as baseline BP, while the previous study used the last recorded MAP value before induction of anesthesia as the baseline BP. Furthermore, intraprocedural mean MAP in our study was relatively higher (87 vs. 76 mmHg). A possible explanation might be that, in the study, the mean cumulated time (in minutes) of a BP drop of 30 mmHg was much less than that of BP drops of 10 mmHg (32.2 vs. 93.3 min). And the difference of mean cumulated time of MAP drops more than 30 mmHg between the good and poor outcome groups was less than that of 10 mmHg group (12.4 vs. 39.2 min). It further revealed that clinicians should not only pay attention to the extent of BP drops but also to the duration of BP drops during procedures, which might have a greater impact on outcomes than BP drops alone.

The major difference between our study and other previous studies is that our observation index is the duration of BP drops, not merely the extent of BP drops. Theoretically, BP elevation may be a natural response of the organism to persistent vessel occlusion in the acute ischemic stroke phase, in order to increase the perfusion of salvageable tissue and to minimize the ischemic damage (26, 27). Maintaining BP at a relatively high level may be the compensatory mechanism and important for LVO patients before achieving successful recanalization. Therefore, one could posit that prolonged episodes of BP drop during general anesthesia might compromise collateral flow, worsen the extent of injury to the ischemic penumbra, and lead to complete infarction. And it may also, to some extent, reflect that the impairment of cerebral dynamic autoregulation is more severe. It is thus conceivable that prolonged episodes of BP drop during general anesthesia may be potentially deleterious. These findings emphasize the need for a close monitoring of BP during general anesthesia. It seems reasonable to minimize the duration of anesthesia induced hypotension during MT. Future guidelines on BP management during MT should take into account not only the BP drop threshold but also the duration of BP drops.

Limitations include the study being performed in a single center and with small number of cases. We collected data prospectively, which has the potential of selection bias. Second, we recorded the BP values only every five min during the procedure. It would be more informative to record BP every 2 or 3 min or even every min. Third, the site of LVO occlusion may also have played a role in the bias of the hemodynamic data as BP pathophysiology and optimal approach of management could be largely different between anterior and posterior circulation strokes. Fourth, inclusion criteria provide heterogeneity in baseline profiles of patients. We enrolled LVO patients within 6 h of symptom onset between January 2018 and April 2018. Afterwards, patients presenting at the hospital 6–16 h after symptom onset were also included according to the criteria of DEFUSE-3 and DAWN trial. Fifth, we did not explore the impact of different anesthetic drugs and doses on BP and clinical outcome, which are worthy of further study. Sixth, we had no standardized protocol for general anesthesia or intubation, which may lead to selection bias.

## Conclusion

Prolonged episodes of intraprocedural MAP lowering were more likely to have poor outcome in LVO patients following MT with general anesthesia. Every 1 min extension in duration of ΔMAP more than 10 and 15 mmHg before recanalization increased the odds of poor outcome by an estimated 1.3 and 1.1%, respectively. Larger prospective studies are warranted to confirm our results and give further insights in periprocedural BP management of AIS patients.

## Data Availability Statement

The raw data supporting the conclusions of this article will be made available by the authors, without undue reservation.

## Ethics Statement

The studies involving human participants were reviewed and approved by the Human Ethics Committee of Zhejiang Provincial People's Hospital. The patients/participants provided their written informed consent to participate in this study.

## Author Contributions

CX: substantial contributions to study design, data collection for the whole trial, data analysis, interpretation of data, and drafting and revising the manuscript for intellectual content. GL: substantial contributions to study design, cleaning and analyzing the data, and revising the manuscript for intellectual content. ZZ, TJ, and NL: data acquisition, interpretation of data, and revising the manuscript for intellectual content. HM: intraprocedural data collection for the whole trial and revising the manuscript for intellectual content. SY: image data collection for the whole trial and revising the manuscript for intellectual content. ZY: writing the statistical analysis plan. YG: monitoring data collection for the whole trial and revising the manuscript critically for intellectual content. ZS: developing study concept/design, study supervision, interpretation of data, revising the manuscript critically for intellectual content, and final approval of the version to be published. All authors agree to be accountable for all aspects of the work in ensuring that questions related to the accuracy or integrity of any part of the work are appropriately investigated and resolved.

## Conflict of Interest

The authors declare that the research was conducted in the absence of any commercial or financial relationships that could be construed as a potential conflict of interest.
